# Nondestructive Determination of Tocopherol and Tocotrienol in Vitamin E Powder Using Near- and Mid-Infrared Spectroscopy

**DOI:** 10.3390/foods13244079

**Published:** 2024-12-17

**Authors:** Saowaluk Rungchang, Sila Kittiwachana, Sujitra Funsueb, Chitsiri Rachtanapun, Juthamas Tantala, Phumon Sookwong, Laichheang Yort, Chayanid Sringarm, Sudarat Jiamyangyuen

**Affiliations:** 1 Department of Agro-Industry, Faculty of Agriculture Natural Resources and Environment, Naresuan University, Phitsanulok 65000, Thailand; saowalukr@nu.ac.th (S.R.); yortl61@nu.ac.th (L.Y.); 2 Department of Chemistry, Faculty of Science, Chiang Mai University, Chiang Mai 50200, Thailand; silacmu@gmail.com (S.K.); sujitra.funs@gmail.com (S.F.); phumon.s@cmu.ac.th (P.S.); 3 Office of Research Administration, Chiang Mai University, Chiang Mai 50200, Thailand; juthamas.t@cmu.ac.th; 4 Department of Food Science and Technology, Faculty of Agro-Industry, Kasetsart University, Bangkok 10900, Thailand; chitsiri.t@ku.th; 5 Department of Agro-Industrial, Food, and Environmental Technology, Faculty of Applied Science, King Mongkut’s University of Technology North Bangkok (KMUTNB), Bangkok 10800, Thailand; 6 Division of Food Science and Technology, Faculty of Agro-Industry, Chiang Mai University, Chiang Mai 50100, Thailand

**Keywords:** tocopherol, tocotrienol, near-infrared spectroscopy, mid-infrared spectroscopy, chemometrics

## Abstract

Vitamin E is an essential nutrient, but its poor water solubility limits food and pharmaceutical applications. The usability of vitamin E can be enhanced via modification methods such as encapsulation, which transforms the physical state of vitamin E from a liquid to a powder. This study examined the efficacy of near-infrared (NIR) and mid-infrared (MIR) spectroscopy in identifying and predicting various vitamin E derivatives in vitamin E-encapsulated powder (VEP). An MIR analysis revealed the fundamental C–H vibrations of vitamin E in the range of 2700–3250 cm^−1^, whereas an NIR analysis provided information about the corresponding combination, first, and second overtones in the range of 4000–9000 cm^−1^. The MIR and NIR data were analyzed using a principal component analysis to characterize the VEP. Partial least squares (PLS) regression was applied to predict the content of individual vitamin E derivatives. PLS cross-validation revealed that NIR analysis provides more reliable predictive accuracy and precision for the contents of vitamin E derivatives, achieving a higher coefficient of determination for prediction (Q^2^) (0.92–0.99) than MIR analysis (0.20–0.85). For test set validation, the NIR predictions exhibited a significant level of accuracy, as indicated by a high ratio of prediction to deviation (RPD) and Q^2^. Furthermore, the PLS models developed using the NIR data had statistically significant predictive performance, with a high RPD (1.54–3.92) and Q^2^ (0.66–0.94). Thus, NIR spectroscopy is a valuable nondestructive technique for analyzing vitamin E samples, while MIR spectroscopy serves as a useful method for confirming its presence.

## 1. Introduction

Vitamin E or tocols is the collective name for a group of fat-soluble compounds found in plants. These compounds have structures characterized by a chromanol ring with a side chain at the C2 position. Vitamin E encompasses eight distinct molecules, including α-, β-, γ-, and δ-tocopherols (Toc) and the four corresponding tocotrienols (T3). Toc possesses a saturated phytyl side chain, whereas T3 has an unsaturated isoprenyl side chain with double bonds at the C3′, C7′, and C11′ positions, with those at the C3′ and C7′ positions having trans configurations. The α-, β-, γ-, and δ-isoforms vary in terms of the quantity and arrangement of methyl groups on the chromanol ring. α-Toc and α-T3 have methyl groups at the C5, C7, and C8 positions of the chromanol ring. In contrast, the β- and γ-isoforms include two methyl groups, and the δ-isoforms possess only one methyl group [1].

Seeds, cereals, and vegetable oils are the primary natural sources of vitamin E. The distillates of vegetable oil, a byproduct of the refining process, are widely employed as the primary source for commercial production [2]. Rice bran oil deodorizer distillate (RBODD) is less common in the food sector than soybean oil and palm oil distillates, mostly because the latter oils are manufactured on a larger scale. RBODD is typically utilized as animal feed; however, owing to its high content of bioactive components, particularly tocotrienol, this byproduct is currently of interest as a vitamin E source. Because of its water insolubility, vitamin E requires modification to overcome its limited utility for food and pharmaceutical applications [3,4,5]. Encapsulation is a notable approach for enhancing the water solubilization of oil-soluble vitamins [6]. Several cell wall components, including maltodextrin, gum arabic (GA), sodium caseinate, pectin, starch, chitosan, and whey proteins, have been used for encapsulation. In particular, GA and maltodextrin are commonly used for encapsulation applications. Maltodextrins are characterized as having a dextrose equivalent (DE) of less than 20. The DE value can be utilized as a quantitative measure of the concentration of glucose molecules in maltodextrin [7,8,9].

Vitamin E can be efficiently identified in various liquids, such as oils, serum, human milk, and food, using diverse analytical techniques, including ultraviolet-visible (UV-Vis) spectroscopy, high-performance liquid chromatography (HPLC), infrared spectroscopy, and spectrophotometric approaches. Recently, several techniques have been developed to analyze the presence of tocols in cosmetics and food products [10]. Near-infrared (NIR) and mid-infrared (MIR) spectroscopy allow for the rapid and nondestructive analysis of different types of samples. Moreover, minimal sample preparation is required. These techniques provide detailed chemical data that can be used in quality control, environmental monitoring, and agricultural management. Owing to their adaptability, speed, and cost-effectiveness, these technologies have become essential in various industries.

NIR spectroscopy encompasses the wavenumber range of 4000–14,000 cm^−1^, which mainly includes the overtones and combinations of fundamental vibrations originating from C–H, N–H, O–H, and S–H bonds [11,12,13]. In contrast, MIR spectroscopy in the wavenumber range of 400–4000 cm^−1^ is mostly associated with the fundamental vibrations, including stretching, bending, and rotating, of certain covalent bonds [14,15]. MIR spectra exhibit more distinct absorption bands than NIR spectra, which tend to be complicated by the presence of multiple overlapping peaks. Previous studies have shown the efficacy of NIR and/or MIR spectroscopy for discrimination by using principal component analysis (PCA) to classify olive pomace cultivars. Notably, this study demonstrated the ability of these techniques to detect the C–H bonds of tocopherol in olive pomace [15,16]. Chemometrics, which is an analytical discipline focused on mining meaningful information from data, is essential for MIR and NIR analyses. In particular, partial least squares (PLS) regression establishes a connection between the actual and predictive data [17]. Furthermore, PCA can be utilized to analyze data formation and as a classification tool [18,19].

The main objective of this study was to investigate the effectiveness of NIR and MIR spectroscopy for identifying and predicting different vitamin E derivatives in encapsulated vitamin E powder (VEP).

## 2. Materials and Methods

### 2.1. Materials

RBODD was provided by Surin Bran Oil Co., Ltd. (Surin, Thailand). Tapioca starch (DE-0), purchased from Bangkok Inter Food Co., Ltd. (Bangkok, Thailand), was used as a control. Maltodextrin samples with various DE values (DE-7, DE-10, and DE-16) were supplied by WGC Co., Ltd. (Nakhon Pathom, Thailand). GA was purchased from Sigma-Aldrich (Steinheim, Germany).

### 2.2. Sample Preparation

The vitamin E extraction method has been previously described [20]. Vitamin E was extracted from RBODD using ethanol at a ratio of 1:9.5 (*w*/*v*). The extraction mixture was refluxed at 80 ± 5 °C for 30 min and then incubated at −26 °C for 24 h. The ethanol was evaporated at 40 °C under vacuum conditions using a rotary evaporator to collect the vitamin E extract (VEE), which was stored in amber glass vials at −20 °C until use.

Encapsulation was performed using a previous method with some modifications [20,21]. The RBODD-derived VEE was used as the core material in the encapsulation process. A 4 × 3 factorial design was used, with four DE values (0, 7, 10, and 16) and three ultrasonic emulsification times (UT; 0, 30, and 60 min). Each of the 12 treatments was conducted in triplicate. A GA/DE/water/VEE ratio of 1:1:4:2 was used for encapsulation. The mixture was placed in a 150 W ultrasonic bath at 37 kHz (Elmasonic S 60 H, Elma Schmidbauer GmbH, Singen, Germany) for the required emulsification time. The emulsion was then frozen at −40 °C for 18 h and lyophilized at −80 °C for 24 h. The resulting encapsulated vitamin E was ground using a blender (model HGB2WT, Waring Commercial, Stamford, CT, USA) to obtain a powder, which was passed through a 20 mesh (850 μm) sieve and collected in an aluminum vacuum bag until analysis. The final samples were denoted as vitamin E-encapsulated powder (VEP).

### 2.3. NIR and MIR Spectroscopic Analysis

NIR spectroscopy was performed using a Matrix-F Fourier transform NIR spectrometer (Bruker Fibre Optics, Ettlingen, Germany). The instrument was fitted with a 1.0 m fiber optic scattered reflectance probe and a thermoelectrically cooled InGaAs detector. The fiber optic cable gathers light scattered in various directions and directs it toward the spectrometer. The NIR spectra of the VEP samples were collected in the reflectance mode in the wavenumber range of 4000–12000 cm^−1^ at a spectral resolution of 16 cm^−1^ with 32 scans. For the NIR measurements, 150 g of each VEP sample was placed in a quartz cup [100 mm (Ø) × 20 mm (H)]. The NIR absorbance was used to facilitate the subsequent data analysis.

After the NIR analysis, 5 mg of the VEP sample was used for the MIR analysis (Spectrum GX, PerkinElmer, Ventura, CA, USA). The MIR spectra were recorded in the wavenumber range of 400–4000 cm^−1^ at a spectral resolution of 4 cm^−1^. For the MIR spectroscopy, absorbance was also used to facilitate the data analysis.

### 2.4. Vitamin E Determination

Tocols were analyzed using an Agilent HPLC 1100 apparatus equipped with a VertiSep™ UPS Silica column (4.6 × 250 mm, 5 μm; Vertical Chromatography Co., Ltd., Nonthaburi, Thailand) and a fluorescence detector (Model 1046A; Hewlett Packard, Bangalore, IN, USA). The mobile phase was a mixture of hexane, tetrahydrofuran, and isopropanol (93:6:1), and elution was isocratic at a flow rate of 0.5 mL/min. The column was held at a constant temperature of 30 °C. Tocols were detected using fluorescence with excitation at 294 nm and emission at 326 nm. The samples were prepared by diluting 1 g of VEP in 1.00 mL of dichloromethane. The solution was filtered using a nylon syringe filter (pore size: 0.45 μm). The sample injection volume was 5.0 μL.

### 2.5. Data Analysis

PCA was used to analyze the MIR and NIR data based on the corresponding differences in the DE values of the encapsulated vitamin E. After applying data preparation techniques, the PCA scores of the first principal component (PC1) were used to identify the samples that deviated from the others, potentially due to variations in DE [22,23].

The MIR and NIR data were used to create a PLS model [23,24]. Cross-validation techniques were employed to develop models for both the MIR and NIR data. However, test set validation was only employed to model the NIR data. The samples were divided into calibration and test sets in a ratio of 2:1 using the Kennard Stone algorithm [25]. The samples were arranged in ascending order according to their modeling responses, ranging from minimum to maximum. The test set comprised alternate samples from a set of three samples. Several statistical metrics were computed to assess the efficacy of the prediction models. The quality prediction of the training set or auto-prediction was evaluated using the coefficient of determination for calibration (R^2^) and root mean square error of calibration (RMSEC). The performance of the models in predicting the test sets or unknown samples was assessed by calculating the coefficient of determination for prediction (Q^2^) and root mean square error of prediction (RMSEP). Furthermore, standard deviation (SD) was employed to examine the variability of the response values. Additionally, root mean square error (RMSE) was used to quantify the accuracy of the predictions. These measures were then combined to calculate the ratio of prediction to deviation (RPD), which served as a standardized metric for evaluating the predictive accuracy. The calibration models were optimized using a leave-one-out cross-validation approach. The number of latent variables (LVs) for each model was selected as the one providing the minimum RMSEC and RMSECV [26].

The chemometric computations and statistical analyses were performed using in-house MATLAB scripts (MATLAB V7.0, The Math Works Inc., Natick, MA, USA). The subtraction of the MIR and NIR spectral data was performed using the OPUS version 7.8 software (Bruker Optics).

## 3. Results and Discussion

### 3.1. Characterization of NIR and MIR Spectral Data

**Figure 1:**
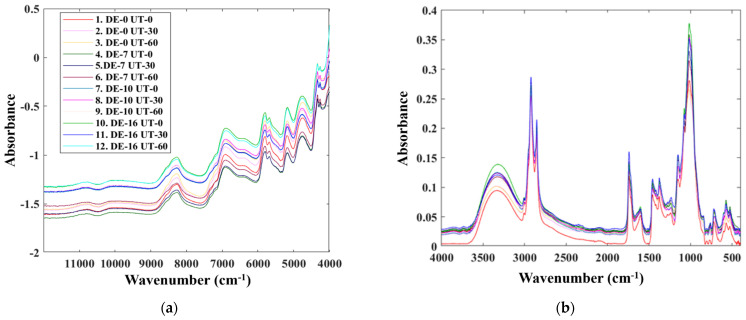
(**a**) NIR and (**b**) MIR spectra of encapsulated vitamin E.

Although vitamin E has previously been characterized using NIR and MIR spectroscopy, this is the first study in which combined NIR and MIR spectral analyses were applied to identify different vitamin E derivatives in VEP. The NIR and MIR spectra of the encapsulated vitamin E are shown in Figure 1. The NIR spectra exhibit multiple overlapping bands owing to a combination of the first and second overtones and fundamental vibrations, primarily involving C–H bonds. A wide absorption peak appears at 8275 cm^−1^, likely caused by the second overtones of CH and CH=CH– stretching vibrations from the oil (Figure 1a). A high-intensity absorption peak is also observed around 5800 cm^−1^, which is associated with the first overtone of CH. The intense absorption peak at approximately 4347 cm^−1^ originates from a combination of the fundamental vibrations of CH groups. This observation is consistent with the findings of an earlier study [24]. The spectra also exhibit intense bands corresponding to water, mostly at 7142–6667 cm^−1^ for the first overtone and 5319–4517 cm^−1^ for the combination band. These bands overlap with the first overtone of the CH vibrations of various chemical groups (CH_3_ and CH_2_). For the latter spectral band, it should be noted that the primary component of oils and triglycerides is triolein, which exhibits a prominent absorption peak at 5800 cm^−1^, representing a distinctive feature of oils, as previously reported by Cayuela and García [16]. A prominent peak corresponding to the first overtone of CH is evident at 5800 cm^−1^ along with a combination band in the range of 5319–4762 cm^−1^. Additionally, a strong absorbance peak appears at 4347 cm^−1^, resulting from the combined fundamental vibrations of the CH groups. Spectral characteristics can offer useful insights into the chemical compositions of oils, indicating the presence of triglycerides such as triolein. The number and position of the absorption peaks can aid in identifying and quantifying various types of oils. Furthermore, infrared spectra can be used to reveal the presence of additional functional groups in oils, including esters and aldehydes. Consequently, infrared spectroscopy is a valuable technique for oil analysis and quality assurance in diverse sectors, such as food and pharmaceuticals.

Figure 1b shows the MIR spectra of the encapsulated vitamin E within the range of 400–4000 cm^−1^, which is referred to as the fundamental infrared region. The stretching of single bonds is observed within the range of 2500–4000 cm^−1^, with the stretching of C–H bonds appearing within the range of 2700–3250 cm^−1^. The characteristic features of vitamin E appear in this region, and the presence of encapsulated vitamin E can be verified using the same region. The C–H alkane groups of vitamin E appear at 2925 cm^−1^, which is a fundamental vibration of vitamin E (Toc and T3). This finding is consistent with previous results [27] and confirms the presence of peaks corresponding to encapsulated vitamin E in the same region. Thus, infrared spectroscopy can contribute to a better understanding of the molecular composition and characteristics of vitamin E.

These results indicate that the presence of vitamin E, an oil-soluble compound, can be effectively detected using both NIR and MIR spectroscopy [28,29]. NIR spectroscopy primarily detects the oil content of vitamin E based on the second, first, and combination overtones, whereas MIR spectroscopy detects fundamental vibrations. The combination of these two techniques provides a comprehensive analysis of vitamin E, allowing for accurate and reliable results. Additionally, NIR spectroscopy offers the advantages of being nondestructive and faster than MIR spectroscopy. Overall, the use of both NIR and MIR spectroscopic techniques can enhance the efficiency and sensitivity of vitamin E detection in various applications, such as food quality control and pharmaceutical analysis. Thus, data from both methods can be used to evaluate encapsulated vitamin E samples and effectively predict the concentrations of vitamin E derivatives using real chemical analysis data, thereby providing a more comprehensive understanding of the samples and their components. Moreover, the complementary nature of the NIR and MIR spectroscopic techniques allows for more robust and accurate analyses. The integration of these techniques into a vitamin E analysis will ensure the reliability and precision of the results, ultimately benefiting industries that rely on accurate detection for quality control and regulatory compliance.

### 3.2. Exploratory Data Analysis of NIR and MIR Spectra Using PCA

**Figure 2:**
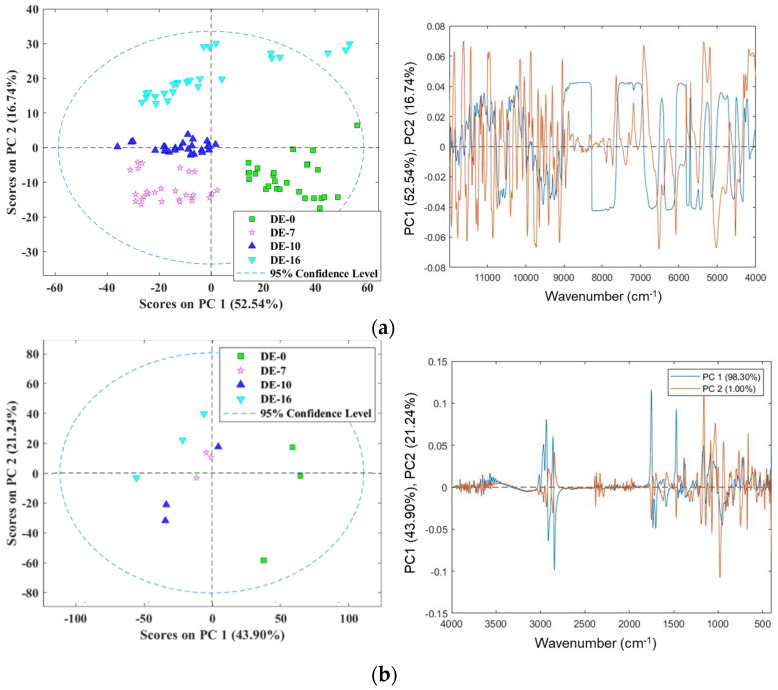
PCA score and loading plots of (**a**) NIR and (**b**) MIR data for encapsulated vitamin E.

PCA score plots were employed to analyze the encapsulated vitamin E samples prepared using DE-0, DE-7, DE-10, and DE-16. For both the NIR and MIR data, the first derivative with mean centering was used for data preprocessing before conducting PCA. This approach was chosen to facilitate the exploration of the inherent characteristics of the raw spectra obtained from samples with various DE values. Following data preparation, PCA was applied to verify the presence of clusters based on DE. For this analysis, the NIR and MIR spectra were analyzed individually. Differences based on the DE value became more evident following the application of PCA to the data, as illustrated in Figure 2. For the MIR data, clear differences were observed for DE-0 and DE-16 compared with DE-7 and DE-10, which did not display such differentiation (Figure 2b). This phenomenon can be attributed to the PCA score plot, in which PC1 and PC2 explained 43.90% and 21.24% of the total variance, respectively. Collectively, these two principal components accounted for 65.14% of the variation in the MIR data. For the NIR data, 69.28% of the variation could be accounted for by PC1 and PC2 (52.54% and 16.74%, respectively; Figure 2a). Moreover, the NIR data allowed the separate identification of DE-0, DE-7, DE-10, and DE-16. The loading plots for the NIR and MIR data are interpreted regarding the encapsulated vitamin E samples based on the different DE levels. The wavenumber regions from 4390 to 4472 cm^−1^ contribute to the DE value, and the absorbance band in the range of 5550–6250 cm^−1^ can be attributed to the first overtone region of carbohydrates (C–H bands) [30,31,32,33,34]. The spectral range of interest, 4000–9000 cm^−1^, exhibits absorption bands associated with CH, CH_2_, and CH_3_ functional groups in vitamin E. For the MIR data, the peak at 900–1200 cm^−1^ and 1200–1500 cm^−1^ can be associated with the C-H group and C-O bonds, which may be related to carbohydrates and vitamin E [35,36,37]. The absorbance within the range of 2700–3200 cm^−1^ corresponds to the stretching of C–H bonds in vitamin E [27,35].

### 3.3. Distribution of Calibration and Validation Reference Data for MIR and NIR Prediction Models

The calibration model used the reference data presented in Table 1. The reference data for each parameter showed a normal distribution based on the mean. A cross-validation approach was employed to predict the models for both the MIR spectroscopy (12 samples) and NIR spectroscopy (108 samples). However, test set validation was only performed for the NIR spectroscopy, with the calibration and validation sets consisting of 72 and 36 samples, respectively. The parameters for the cross-validation of the α-Toc, β-Toc, γ-Toc, δ-Toc, Toc, α-T3, γ-T3, δ-T3, T3, and tocol data are shown in Table 1. In addition to being used for cross-validation, these datasets were applied as calibration sets in test set validation for the NIR spectroscopy. The validation datasets for the test set validation of these parameters are also shown in Table 1. When evaluating the descriptive statistics, the samples exhibited a significant variation in the derivative values of Toc, T3, and tocols. Moreover, the external validation set was within the limits of the calibration set. These results correspond with those of the previous studies [11,16,36] that utilized NIR spectroscopy to analyze tocopherol compounds.

### 3.4. Quantitative Analysis of Encapsulated Vitamin E Using PLS Regression

#### 3.4.1. Cross-Validation of MIR and NIR Predictions

In this study, PLS regression models were established to predict the various forms of vitamin E, including α-Toc, β-Toc, γ-Toc, δ-Toc, Toc, α-T3, γ-T3, δ-T3, T3, and tocols. These models were developed using the data collected from the MIR and NIR spectra (X) of the encapsulated vitamin E, as well as the corresponding quantitative results (Y) obtained from the reference analyses (Table 1). Table 2 presents the outcomes of the PLS regression on the quality parameters determined from the reference, employing both MIR and NIR predictions. The evaluation included cross-validation and test set validation, with the test set validation mainly applicable to NIR predictions. For cross-validation, the RMSECV and Q^2^ values indicate that MIR analysis provides less accurate predictions than NIR analysis. The Q^2^ values for the MIR analysis ranged from 0.20 to 0.70, whereas those for the NIR analysis ranged from 0.58 to 0.98.

The Q^2^ values for various parameters exhibited notable improvements in predictive accuracy when NIR analysis was used instead of MIR analysis, with increases observed for α-Toc (from 0.70 to 0.98), β-Toc (from 0.52 to 0.93), γ-Toc (from 0.58 to 0.93), δ-Toc (from 0.35 to 0.92), Toc (from 0.64 to 0.96), α-T3 (from 0.20 to 0.58), γ-T3 (from 0.56 to 0.96), δ-T3 (from 0.79 to 0.93), T3 (from 0.58 to 0.96), and tocols (from 0.57 to 0.96). Cross-validation is a self-prediction technique that requires high Q^2^ values.

However, the practical implementation of MIR analysis is insufficient, possibly due to a small number of samples, which results in low prediction accuracy. These findings indicate that the use of NIR spectral data provides a greater level of predictive accuracy. Furthermore, despite using auto prediction, both the MIR and NIR analyses exhibited poor prediction ability for the α-T3 parameters. This phenomenon could be due to the limited dispersion of the SD for α-T3 in the study (Table 1). To overcome this issue and achieve more precise predictions, further studies are required to develop improved approaches and procedures. The NIR data were selected to carry out predictions using test set validation to assess the vitality of the parameters.

#### 3.4.2. Test Set Validation of NIR Predictions

The samples (*n* = 108) were divided into two separate datasets. Calibration models for quality control parameters were developed using a randomly selected set of training samples (*n* = 72). The remaining 36 data samples were used as validation samples to assess the predictive performance of the established models. All the NIR spectra were preprocessed using the first derivative (1st derivative) method, with the exception of δ-Toc, Toc, and tocols, for which the first derivative method was applied followed by the standard normal variate (SNV) approach prior to PLS calibration. Models with high robustness and variability were created by combining the data for all the encapsulated vitamin E samples prepared with DE-0, DE-7, DE-10, and DE-16. The model for each parameter was selected by examining the statistical parameters, including high R^2^, Q^2^, and RPD values and low RMSEC and RMSEP values. Table 2 provides a comprehensive overview of the performance of the built models for all the quality parameters. The R^2^ values for the NIR calibration were high (0.71–0.96), indicating a strong relationship between the predicted and actual values. In addition, the RMSEC values were low, further supporting the accuracy of the calibration model. The regression statistics were strongly influenced by the models developed using the NIR spectra.

According to Pu et al. and Okere et al. [37,38], models exhibiting RPD values below 1.5 are unreliable. Conversely, models with RPD values of 1.5–2.0 have utility for approximate prediction purposes. Furthermore, RPD values of 2.0–2.5 are considered suitable for quantitative predictions, and any RPD values above 3 are satisfactory. In this study, an RPD value above three was observed for α-Toc, Toc, T3, and tocols with Q^2^ = 0.94, 0.92, 0.89, and 0.92, respectively, and RMSEP = 0.56, 1.72, 10.0, and 10.67, respectively. The parameters with RPD values of 1.5–3.0 were β-Toc, γ-Toc, δ-Toc, α-T3, γ-T3 and δ-T3 with Q^2^ values of 0.86, 0.86, 0.82, 0.66, 0.87, and 0.73, respectively, and RMSEP values of 0.16, 1.16, 0.14, 0.22, 10.36, and 0.84, respectively. The observation of the lowest RPD value for α-T3 may be attributable to the same factor as in cross-validation. Specifically, the large range of reference data may result in significant prediction errors when calculating the RPD. These results represent a notable achievement in accurately predicting vitamin E derivatives using the NIR technique, characterized by a high RPD (2.43–3.92), high Q^2^ value (0.82–0.94), and low RMSEP, except for α-T3. In comparison, Cayuela and García [16] previously used NIR spectroscopy and PLS regression to predict the α-Toc (RPD = 2.37), β-Toc (RPD = 1.04), γ-Toc (RPD = 1.91), and Toc (RPD = 2.01) levels in olive oil. In comparison, the current study achieved better RPD values for α-Toc (RPD = 3.92), β-Toc (RPD = 2.56), Toc (RPD = 3.34), and γ-Toc (RPD = 2.60). It should be noted that good results were obtained when the encapsulated vitamin E samples were prepared with DE-0, DE-7, DE-10, and DE-16 in the laboratory room for both the training and test sets. However, these results demonstrate the potential of using NIR for the quantitative analysis of vitamin E content in the samples.

**Figure 3:**
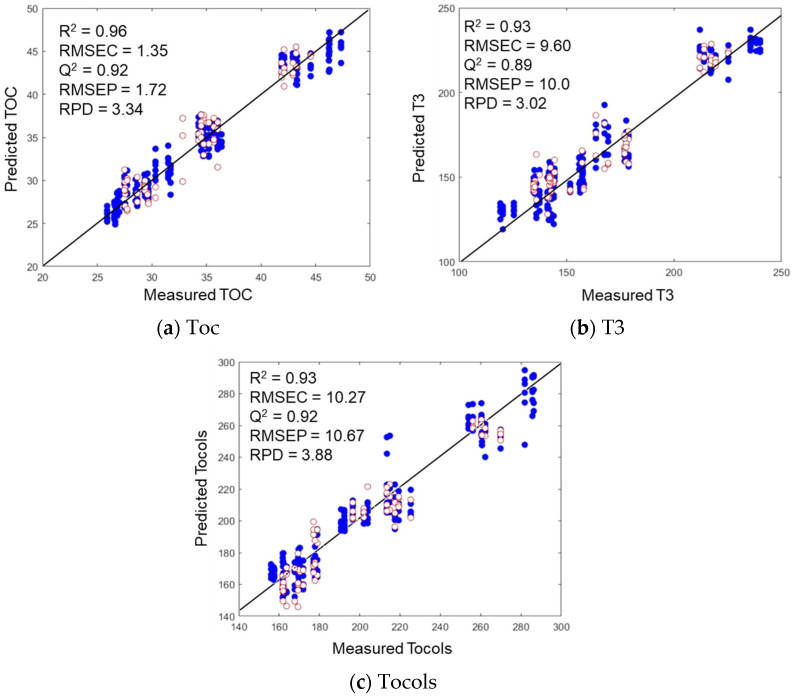
Scatter plots of the actual and predicted quality parameters for the NIR spectra of encapsulated vitamin E.

Figure 3 shows the scatter plots of the quality parameters (Toc, T3, and tocols) obtained from the reference methods and NIR predictions for the encapsulated vitamin E. Significant linearity was observed for the predictions of Toc, T3, and tocols. The calibration models yielded low prediction errors and high coefficients of determination for all these parameters, although the prediction errors were somewhat higher for T3 and tocols than for Toc. When constructing a model with a limited dataset and a significant SD, the associated constraints must be acknowledged and addressed. In particular, small datasets can cause problems such as overfitting and a restricted ability to generalize. However, the tocol parameter, which is characteristic of vitamin E, yielded an RPD of 2.29. This high RPD is suitable for quantitative predictions, as reported by Okere et al. and Kahruman et al. [38,39].

**Figure 4:**
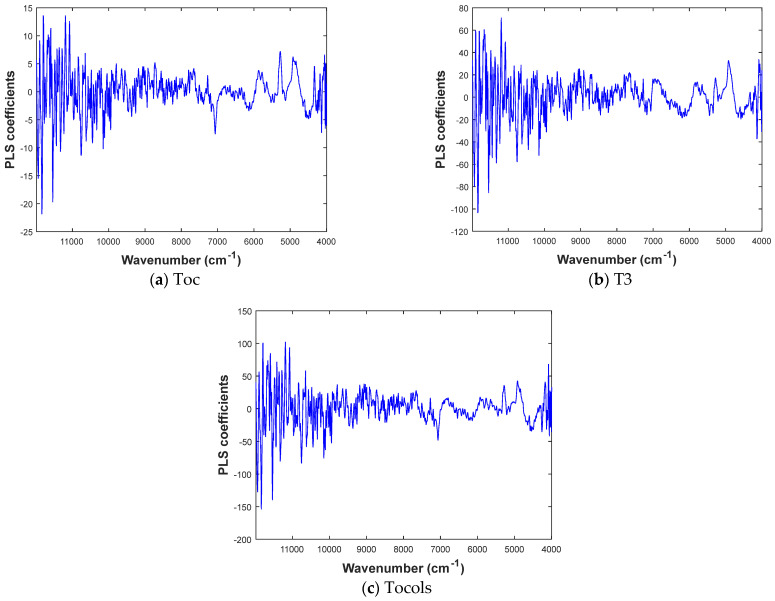
PLS coefficients for the NIR spectra of encapsulated vitamin E.

The PLS regression coefficients associated with the NIR wavenumbers are shown in Figure 4 for Toc, T3, and tocols in the encapsulated vitamin E. Identifying a single significant region is challenging because the values are derived from multiple functional groups. The spectral range of interest (4000–9000 cm^−1^) exhibits absorption bands associated with CH, CH_2_, and CH_3_ functional groups. Moreover, these absorption bands correspond to combination, first, and second overtones, which are known to be significant components of the functional groups in vitamin E. The results presented in this study are consistent with the findings of Antónia Nunes et al. (2020) [15], who observed multiple peaks (4456–4359 cm^−1^) corresponding to the absorption bands of CH_2_ and CH_3_ in tocopherol derived from olive pomace. Similarly, Páscoa et al. [40] identified the CH_2_ and CH_3_ absorption bands in the combination band region (4035–4961 cm^−1^) and the first overtone region (5389–6504 cm^−1^). Moreover, a wide absorption peak appears at 8275 cm^−1^, most likely caused by the second overtones of CH and CH=CH– stretching vibrations from the oil containing vitamin E. These results indicate that the accuracy and reliability of the NIR predictions for identifying different functional groups within vitamin E capsules can be assessed by comparison with other analytical techniques.

## 4. Conclusions

NIR and MIR spectroscopy were demonstrated to be valuable techniques for the analysis of tocopherols and tocotrienols. By analyzing fundamental vibrations using MIR spectroscopy and combination, first, and second overtones using NIR spectroscopy, these approaches provided useful data for interpretation. Nevertheless, the suitability of these methods relies on the attributes of the sample and desired data. Hence, the advantages and constraints of NIR and MIR spectroscopy must be evaluated to determine the optimal technique for specific analytical tasks. NIR spectroscopy is primarily suitable for the accurate prediction of vitamin E contents; however, MIR spectroscopy is more convenient for quality control purposes or for verifying the presence of vitamin E in a sample. Moreover, as a nondestructive technique, NIR spectroscopy is advantageous when the integrity of the sample must be maintained. In contrast, MIR spectroscopy requires sample preparation, which can be time-intensive. Thus, either NIR or MIR spectroscopy may be suitable depending on the analysis requirements.

## Figures and Tables

**Table 1:** Characteristics of sample sets for predicting the calibration model of quality parameters for encapsulated vitamin E.

Parameter	Cross-Validation (MIR: 12 Samples; NIR: 108 Samples)				Test Set Validation (NIR)							
					Calibration Set (*n* = 72)				Validation Set (*n* = 36)			
	Mean	SD	Min	Max	Mean	SD	Min	Max	Mean	SD	Min	Max
α-Toc (mg/g)	12.59	2.70	9.54	16.74	12.68	2.57	9.54	16.74	12.68	2.20	9.68	16.51
β-Toc (mg/g)	2.05	0.53	1.15	2.82	2.03	0.49	1.15	2.82	2.05	0.41	1.32	2.73
γ-Toc (mg/g)	17.11	3.53	12.77	24.29	17.11	3.43	12.41	24.85	17.01	2.86	13.25	24.27
δ-Toc (mg/g)	2.24	0.38	1.63	2.80	2.25	0.37	1.55	2.93	2.23	0.34	1.67	2.81
Toc (mg/g)	33.99	6.94	26.38	46.60	33.99	6.81	25.91	47.33	34.52	5.71	27.50	44.55
α-T3 (mg/g)	1.27	0.39	0.78	2.04	1.26	0.40	0.78	2.04	1.30	0.34	0.81	1.70
γ-T3 (mg/g)	157.71	35.44	115.53	224.15	158.30	35.23	115.53	224.15	156.79	27.70	126.50	212.43
δ-T3 (mg/g)	8.62	2.06	5.43	12.36	8.60	2.01	5.32	12.46	8.81	1.59	5.48	12.37
T3 (mg/g)	167.60	37.62	121.74	238.01	167.24	36.86	119.25	240.08	167.41	30.21	134.73	225.32
Tocols (mg/g)	201.59	44.42	148.12	284.61	199.27	44.65	148.12	284.61	199.34	41.38	161.76	269.87

Toc: tocopherol; T3: tocotrienol; tocols = Toc + T3.

**Table 2:** PLS results for quality parameters of encapsulated vitamin E.

Parameter	Cross-Validation					Test Set Validation					
	NIR		MIR			NIR					
	RMSECV	Q^2^	RMSECV	Q^2^	Preprocessing	LVs	RMSEC	R^2^	RMSEP	Q^2^	RPD
α-Toc (mg/g)	0.37	0.98	1.48	0.70	1st derivative	9	0.55	0.96	0.56	0.94	3.92
β-Toc (mg/g)	0.14	0.93	0.38	0.52	1st derivative	7	0.15	0.90	0.16	0.86	2.56
γ-Toc (mg/g)	0.90	0.93	2.20	0.58	1st derivative	7	1.10	0.89	1.16	0.86	2.60
δ-Toc (mg/g)	0.10	0.92	0.34	0.35	1st derivative + SNV	7	0.13	0.87	0.14	0.82	2.43
Toc (mg/g)	1.26	0.96	4.41	0.64	1st derivative + SNV	8	1.35	0.96	1.72	0.92	3.34
α-T3 (mg/g)	0.25	0.58	0.40	0.20	1st derivative	8	0.20	0.71	0.22	0.66	1.54
γ-T3 (mg/g)	6.85	0.96	22.89	0.56	1st derivative	7	9.31	0.93	10.36	0.87	2.67
δ-T3 (mg/g)	0.54	0.93	0.90	0.79	1st derivative	7	0.71	0.88	0.84	0.73	1.89
T3 (mg/g)	7.41	0.96	23.68	0.58	1st derivative	7	9.60	0.93	10.0	0.89	3.02
Tocols (mg/g)	8.44	0.96	28.20	0.57	1st derivative + SNV	8	10.27	0.93	10.67	0.92	3.88

Cross-validation of the NIR and MIR predictions using standard normal variate (SNV) for data processing.

## Data Availability

The original contributions presented in the study are included in the article; further inquiries can be directed to the corresponding authors.

## References

[1] Niki E., Abe K. (2019). Vitamin E: Structure, Properties and Functions. Vitamin E: Chemistry and Nutritional Benefits.

[2] Ye Z., Shi B., Huang Y., Ma T., Xiang Z., Hu B., Kuang Z., Huang M., Lin X., Tian Z. (2022). Revolution of vitamin E production by starting from microbial fermented farnesene to isophytol. Innovation.

[3] Sawadikiat P., Setwipattanachai P., Chaiseri S., Hongsprabhas P. (2015). Rice phytochemicals concentrated by molecular distillation process and their use as co-surfactant in water dispersion. J. Food Sci. Technol..

[4] Ko S., Lee S., Kim I. (2008). The concentration of tocols from rice bran oil deodorizer distillate using solvent. Eur. J. Lipid Sci. Technol..

[5] Jaiswal S.G., Pradhan S., Patel M., Naik M., Naik S. (2014). Rice Bran Oil Distillate, a Choice for Gamma-Oryzanol: Separation and Oxidative Stability Study. J. Food Res..

[6] Fan C., Feng T., Wang X., Xia S., Swing C.J. (2022). Liposomes for encapsulation of liposoluble vitamins (A, D, E and K): Comparation of loading ability, storage stability and bilayer dynamics. Food Res. Int..

[7] Rayhani Z., Kurniasih E., Savia, Fadhilah R. (2018). Classification of dextrose equivalent analysis maltodextrin starch seeds through enzymatic hydrolysis reaction. IOP Conf. Ser. Mater. Sci. Eng..

[8] Sringarm C., Numthuam S., Jiamyangyuen S., Kittiwachana S., Kielar F., Wongsaipun S., Rungchang S. (2024). Quantitative and qualitative evaluation of maltodextrin products in the industry using near-infrared spectroscopy. Int. J. Food Sci. Technol..

[9] Sringarm C., Numthuam S., Jiamyangyuen S., Kittiwachana S., Saeys W., Rungchang S. (2024). Classification of industrial tapioca starch hydrolysis products based on their Brix and dextrose equivalent values using near-infrared spectroscopy. J. Sci. Food Agric..

[10] Gamna F., Spriano S. (2021). Vitamin E: A Review of Its Application and Methods of Detection When Combined with Implant Biomaterials. Materials.

[11] Katuwal S., Knadel M., Moldrup P., Norgaard T., Greve M.H., de Jonge L.W. (2018). Visible–Near-Infrared Spectroscopy can predict Mass Transport of Dissolved Chemicals through Intact Soil. Sci. Rep..

[12] Medeiros M.L.d.S., Lima A.F., Gonçalves M.C., Godoy H.T., Barbin D.F. (2023). Portable near-infrared (NIR) spectrometer and chemometrics for rapid identification of butter cheese adulteration. Food Chem..

[13] Sringarm C., Numthuam S., Jiamyangyuen S., Klangpetch W., Wongsaipun S., Kittiwachana S., Saeys W., Rungchang S. (2023). Quantification of individual sugars in tapioca syrups with near-infrared spectroscopy. J. Food Compos. Anal..

[14] Huck C.W. (2014). Advances of vibrational spectroscopic methods in phytomics and bioanalysis. J. Pharm. Biomed. Anal..

[15] Nunes M.A., Páscoa R.N., Alves R.C., Costa A.S., Bessada S., Oliveira M.B.P. (2020). Fourier transform near infrared spectroscopy as a tool to discriminate olive wastes: The case of monocultivar pomaces. Waste Manag..

[16] Cayuela J.A., García J.F. (2017). Sorting olive oil based on alpha-tocopherol and total tocopherol content using near-infra-red spectroscopy (NIRS) analysis. J. Food Eng..

[17] Díaz E.O., Kawamura S., Matsuo M., Kato M., Koseki S. (2019). Combined analysis of near-infrared spectra, colour, and physicochemical information of brown rice to develop accurate calibration models for determining amylose content. Food Chem..

[18] Fernández-Espinosa A.J. (2016). Combining PLS regression with portable NIR spectroscopy to on-line monitor quality parameters in intact olives for determining optimal harvesting time. Talanta.

[19] Buvé C., Saeys W., Rasmussen M.A., Neckebroeck B., Hendrickx M., Grauwet T., Van Loey A. (2022). Application of multivariate data analysis for food quality investigations: An example-based review. Food Res. Int..

[20] Yort L., Singanusong R., Yuenyong J., Sookwong P., Jiamyangyuen S. (2022). Optimization of Vitamin E Extraction from Rice Bran Oil Deodorizer Distillate using Response Surface Methodology. Curr. Res. Nutr. Food Sci. J..

[21] Sahlan M., Fadhan A.M., Pratami D.K., Lischer K., Wijanarko A., Hermansyah H., Mahira K.F. (2019). Encapsulation of Agarwood Essential Oil with Maltodextrin and Gum Arabic. Int. J. Technol..

[22] Manley M. (2014). Near-infrared spectroscopy and hyperspectral imaging: Non-destructive analysis of biological materials. Chem. Soc. Rev..

[23] Funsueb S., Thanavanich C., Theanjumpol P., Kittiwachana S. (2023). Development of new fruit quality indices through aggregation of fruit quality parameters and their predictions using near-infrared spectroscopy. Postharvest Biol. Technol..

[24] Sringarm C., Numthuam S., Singanusong R., Jiamyangyuen S., Kittiwatchana S., Funsueb S., Rungchang S. (2022). Quantitative determination of quality control parameters using near infrared spectroscopy and chemometrics in process monitoring of tapioca sweetener production. LWT.

[25] Kennard R.W., Stone L.A. (1969). Computer-aided Design of Experiments. Technometrics.

[26] Marrubini G., Papetti A., Genorini E., Ulrici A. (2016). Determination of the Sugar Content in Commercial Plant Milks by Near Infrared Spectroscopy and Luff-Schoorl Total Glucose Titration. Food Anal. Methods.

[27] Fathi M., Nasrabadi M.N., Varshosaz J. (2017). Characteristics of vitamin E-loaded nanofibres from dextran. Int. J. Food Prop..

[28] Cayuela-Sánchez J.A., Palarea-Albaladejo J., García-Martín J.F., Pérez-Camino M. (2019). del C. Olive Oil Nutritional Labeling by Using Vis/NIR Spectroscopy and Compositional Statistical Methods. Innov. Food Sci. Emerg. Technol..

[29] Oliveira-Folador G., Bicudo M.d.O., de Andrade E.F., Renard C.M.-G.C., Bureau S., de Castilhos F. (2018). Quality traits prediction of the passion fruit pulp using NIR and MIR spectroscopy. LWT.

[30] De Oliveira G.A., de Castilhos F., Renard C.M.G.C., Bureau S. (2014). Comparison of NIR and MIR Spectroscopic Methods for Determination of Individual Sugars, Organic Acids and Carotenoids in Passion Fruit. Food Res..

[31] Bázár G., Romvári R., Szabó A., Somogyi T., Éles V., Tsenkova R. (2016). NIR detection of honey adulteration reveals differences in water spectral pattern. Food Chem..

[32] López M.G., García-González A.S., Franco-Robles E. (2017). Carbohydrate Analysis by NIRS-Chemometrics. Dev. Near-Infrared Spectrosc..

[33] Henn R., Schwab A., Huck C.W. (2016). Evaluation of benchtop versus portable near-infrared spectroscopic method combined with multivariate approaches for the fast and simultaneous quantitative analysis of main sugars in syrup formulations. Food Control..

[34] Masithoh R.E., Amanah H.Z., Yoon W.S., Joshi R., Cho B.K. (2021). Determination of Protein and Glucose of Tuber and Root Flours Using NIR and MIR Spectroscopy. Infrared Phys. Technol..

[35] Borghi F.T., Santos P.C., Santos F.D., Nascimento M.H., Corrêa T., Cesconetto M., Pires A.A., Ribeiro A.V., Lacerda V., Romão W. (2020). Quantification and classification of vegetable oils in extra virgin olive oil samples using a portable near-infrared spectrometer associated with chemometrics. Microchem. J..

[36] Xu J., Nwafor C.C., Shah N., Zhou Y., Zhang C. (2019). Identification of genetic variation in *Brassica napus* seeds for tocopherol content and composition using near-infrared spectroscopy technique. Plant Breed..

[37] Pu Y.-Y., O’donnell C., Tobin J., O’shea N. (2019). Review of near-infrared spectroscopy as a process analytical technology for real-time product monitoring in dairy processing. Int. Dairy J..

[38] Okere E.E., Arendse E., Nieuwoudt H., Perold W.J., Opara U.L. (2022). Non-destructive Evaluation of the Quality Characteristics of Pomegranate Kernel Oil by Fourier Transform Near-Infrared and Mid-Infrared Spectroscopy. Front. Plant Sci..

[39] Kahrıman F., Onaç I., Türk F.M., Öner F., Egesel C. (2019). Determination of carotenoid and tocopherol content in maize flour and oil samples using near-infrared spectroscopy. Spectrosc. Lett..

[40] Páscoa R.N.M.J., Nunes M.A., Reszczyński F., Costa A.S.G., Oliveira M.B.P.P., Alves R.C. (2021). Near Infrared (NIR) Spectroscopy as a Tool to Assess Blends Composition and Discriminate Antioxidant Activity of Olive Pomace Cultivars. Waste Biomass Valorization.

